# 

**Published:** 1896-08

**Authors:** 


					﻿Southern Medical Record.
A Monthly Journal of Medicine and Surgery.
Vol. XXVI.
ATLANTA, GA., AUGUST , 1896.
No. IX.
BERNARD WOLFF, M. D., Editor.
LOUIS H. JONES, M. D., Business Manager.
Associate Editors :
A. W. GRIGGS, M. D.
WILLIS F. WESTMORELAND. M. D.
j. McFadden gaston, m. d.
TERMS: $2.00 Per Annum; Single Copy, 20 Cents.
Contents on Page 6.
PUBLISHED ON THE FIRST OF EACH MONTH.
Entered at the Post Office at Atlanta, Ga., as Second-Class Matter.
The Foote & Davies Co., Atlanta, Ga.
				

